# The 4C5 Cell-Impermeable Anti-HSP90 Antibody with Anti-Cancer Activity, Is Composed of a Single Light Chain Dimer

**DOI:** 10.1371/journal.pone.0023906

**Published:** 2011-09-01

**Authors:** Katerina Sidera, Avraam El Hamidieh, Avgi Mamalaki, Evangelia Patsavoudi

**Affiliations:** 1 Department of Biochemistry, Hellenic Pasteur Institute, Athens, Greece; 2 Department of Medical Instruments Technology, Technological Educational Institute of Athens, Athens, Greece; MRC National Institute for Medical Research, United Kingdom

## Abstract

MAb 4C5 is a cell impermeable, anti-HSP90 murine monoclonal antibody, originally produced using hybridoma technology. We have previously shown that mAb 4C5 specifically recognizes both the α- and to a lesser extent the β-isoform of HSP90. Additionally, *in vitro* and *in vivo* studies revealed that by selectively inhibiting the function of cell-surface HSP90, mAb 4C5 significantly impairs cancer cell invasion and metastasis. Here we describe the reconstitution of mAb 4C5 into a mouse-human chimera. More importantly we report that mAb 4C5 and consequently its chimeric counterpart are completely devoid of heavy chain and consist only of a functional kappa light chain dimer. The chimeric antibody is shown to retain the original antibody's specificity and functional properties. Thus it is capable of inhibiting the function of surface HSP90, leading to reduced cancer cell invasion *in vitro*. Finally, we present *in vivo* evidence showing that the chimeric 4C5 significantly inhibits the metastatic deposit formation of MDA-MB-453 cells into the lungs of SCID mice. These data suggest that a chimeric kappa light chain antibody could be potentially used as an anti-cancer agent, thereby introducing a novel type of antibody fragment, with reduced possible adverse immunogenic effects, into cancer therapeutics.

## Introduction

Heat shock protein 90 (HSP90) is considered a very attractive drug-target for cancer therapy, since most of its client proteins play key roles in the acquisition and/or maintenance of the malignant phenotype [1]–[4]. Recently, we and others have identified a pool of HSP90 at the cell surface [5]–[8], where it was shown to participate in cancer cell invasion and metastasis [9]–[11]. Increasing evidence continues to reinforce the notion of a wide-ranging phenomenon of extracellular HSP90 chaperoning, implicated in cancer progression and metastatic spread [12]–[16] thus supporting the development of inhibitors that specifically target the cell surface HSP90. MAb 4C5 is a cell-impermeable murine monoclonal antibody produced using hybridoma technology [17], that specifically recognizes both the α and to a lesser extent the β isoform of HSP90 [8]. MAb 4C5 was initially shown to inhibit cell migration processes *in vitro* during development of the nervous system [18], [19] by affecting actin cytoskeletal re-arrangement and formation of motile structures such as lamellipodia [8], [20]. Subsequently evidence was presented showing that by binding selectively to the surface pool of HSP90, mAb 4C5 significantly reduces melanoma cell invasion and metastasis [11]. Furthermore mAb 4C5 was shown to inhibit the extracellular interaction between HSP90 and the growth factor receptor ErbB-2 in MDA-MB-453 breast cancer cells, leading to impaired downstream signalling and reduced cancer cell motility and invasion [21]. Finally, mAb 4C5 was shown to inhibit a functional interaction between secreted HSP90 and the inactive forms of metalloproteinases 2 and 9, necessary for the enzymes' activation which is essential for cancer cell invasion and extravasation [14]. These combined data suggested that the unique capacity of mAb 4C5 to specifically inhibit the extracellular pool of HSP90 without affecting the wide range of important intracellular roles of this chaperone could have clinical benefits in the treatment of human malignancies. However, murine mAbs do not constitute ideal therapeutic agents, since their potential immunogenicity represents a limitation to their clinical use. The application of mouse mAbs to human therapy has become feasible by the advent of recombinant DNA technologies which has led to the development of chimeric and humanized antibodies which exhibit reduced immunogenicity [22] without a significant loss in the affinity, especially in the case of chimeric antibodies [23], [24].

In the current work we describe the cloning and sequencing of the mAb 4C5 genes from the originating hybridoma cell line and the successful construction of a functional mouse-human chimera, which is shown to retain the properties of the parental antibody. More importantly we report that mAb 4C5 is completely devoid of heavy (H)-chain and consists of only a functional immunoglobulin kappa light chain dimer and its properties can be recapitulated in a recombinant protein containing only this light (L)-chain polypeptide. Finally, we demonstrate the potential therapeutic efficacy of this novel type of antibody fragment.

## Results

### MAb 4C5 is an antibody fragment completely devoid of a heavy chain

**Figure 1:**
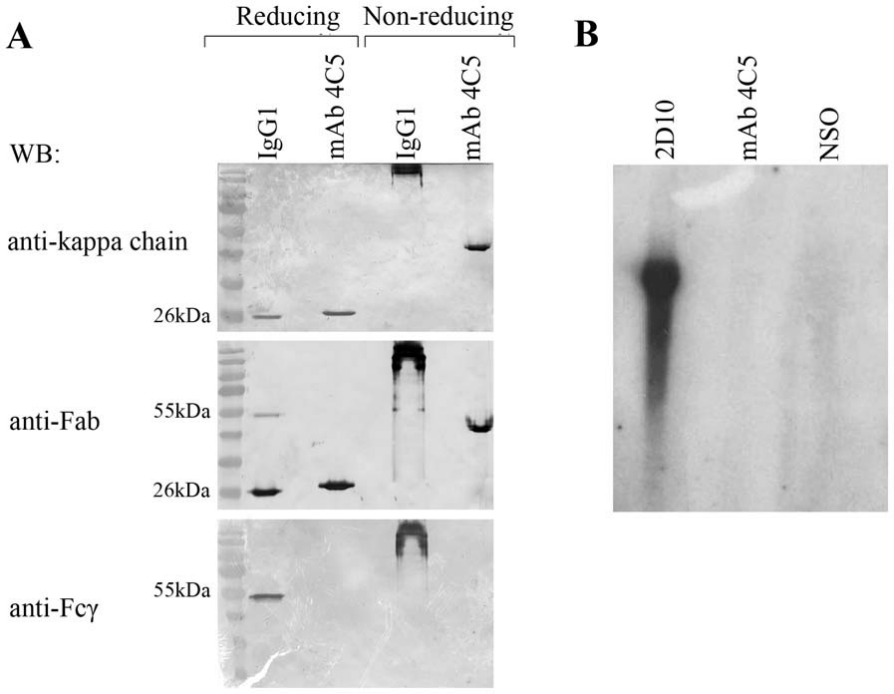
mAb 4C5 is a kappa L- chain dimer. **A**. Electrophoretic analysis of mAb 4C5, followed by immunoblotting with an anti-mouse kappa chain, an anti-mouse Fab and an anti-Fcγ antibody. Intact IgG1 immunoglobulin produced by the 2D10 hybridoma cells serves as positive control. Under reducing electrophoresis followed by western blot with the anti-Fab antibody, a single 25 kDa-immunoreactive band is observed, instead of the 25- and 50-kDa bands corresponding to the L- and H-chain, respectively, of an intact IgG1. This 25 kDa band is identical to the band corresponding to the kappa L-chain as shown by western blot with the anti-mouse kappa chain antibody. Under-non reducing electrophoresis followed by western blot with both the anti-kappa and the anti-Fab antibodies, mAb 4C5 is shown to migrate at approximately 50 kDa, and not at 150 kDa as expected for an intact IgG1 molecule. No mAb 4C5 immunoreactivity is detected after electrophoresis under both reducing and non-reducing conditions followed by western blot with an anti-Fcγ antibody. In the case of the IgG1 immunoglobulin under the same conditions a 50 kDa and a 150 kDa band is observed, respectively. **B**. No radioactivity is detected after northern blot analysis of 4C5 hybridoma-derived RNA with a H-chain radiolabelled probe. RNA derived from the IgG1-producing 2D10 hybridoma cells and the NSO myeloma cells served as positive and negative control, respectively.

The electrophoretic motility of mAb 4C5 studied under reducing and non-reducing SDS-PAGE revealed that it is not a conventional IgG molecule. More specifically, when purified mAb 4C5 was subjected to reducing SDS-PAGE, followed by immunoblotting with an anti-Fab, we did not observe the typical 25 kDa and 50 kDa bands corresponding to the L- and H-chain, respectively of a conventional IgG antibody, but instead a single band at approximately 25 kDa (Fig. 1A). Interestingly an identical 25 kDa band was obtained after immunoblotting with an anti-kappa L-chain antibody (Fig. 1A). Accordingly, after non-reducing electrophoresis immunoblotting with both of these standard-antibodies, shows mAb 4C5 to be significantly smaller than a conventional IgG1 molecule since it migrated at approximately 50 kDa. (Fig. 1A). Finally, no immunoreactivity was detected after electrophoresis of mAb 4C5 under both reducing and non-reducing conditions, followed by immunoblotting using an anti-Fcγ (Fig. 1A). These combined data indicated that mAb 4C5 may either lack a part of its H-chain, or that it is completely devoid of H-chain. In order to further explore these possibilities we next performed northern blot analysis using an IgG1 H-chain probe. RNA derived from hybridoma cells that produce an intact IgG1 immunoglobulin named 2D10 served as positive control. In contrast to the positive control, no radioactivity was detected after hybridization of the RNAs derived from the mAb 4C5 hybridoma and NSO myeloma cells (negative control) (Fig. 1B), indicating that mAb 4C5 may completely lack a H-chain gene. This was further confirmed by H-chain PCR amplification experiments. For the amplification of the H-chain cDNA of mAb 4C5, a panel of eight mouse universal primers and a polyA+ primer respectively directed against the 5′and the 3′ ends of the mRNA, were tested in several separate PCR reactions. In all conditions tested no amplification of a specific H-chain product was observed (data not shown). These combined data suggested that mAb 4C5 is devoid of H-chain and we therefore proceeded to the recombinant expression of the antibody L-chain alone in order to explore its properties.

### Construction of the chimeric L- chain antibody

**Figure 2:**
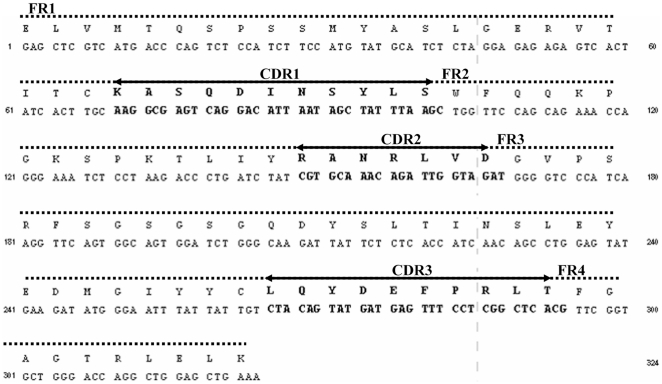
Nucleotide and aminoacid sequences of mAb 4C5. Nucleotide and amino acid sequences of the L-chain variable region of mAb 4C5.

The initial amplification of the mAb 4C5 kappa chain gene from the first strand cDNA template was performed using universal mouse L-chain primers according to Barbas [25]. The PCR product corresponding to the full length mab 4C5 L-chain, was then subcloned into the pComb3H vector. Sequence analysis of recombinant L-chain (rec-4C5) revealed that it belongs to the kappa chain subgroup I [26] (Fig. 2).

Chimeric L-chain antibody (ch-4C5) was constructed by replacing the mouse LCκ coding region with the corresponding human LCκ insert. The sequence analysis of several recombinant clones confirmed the successful construction of the chimeric mouse-human antibody.

**Figure 3:**
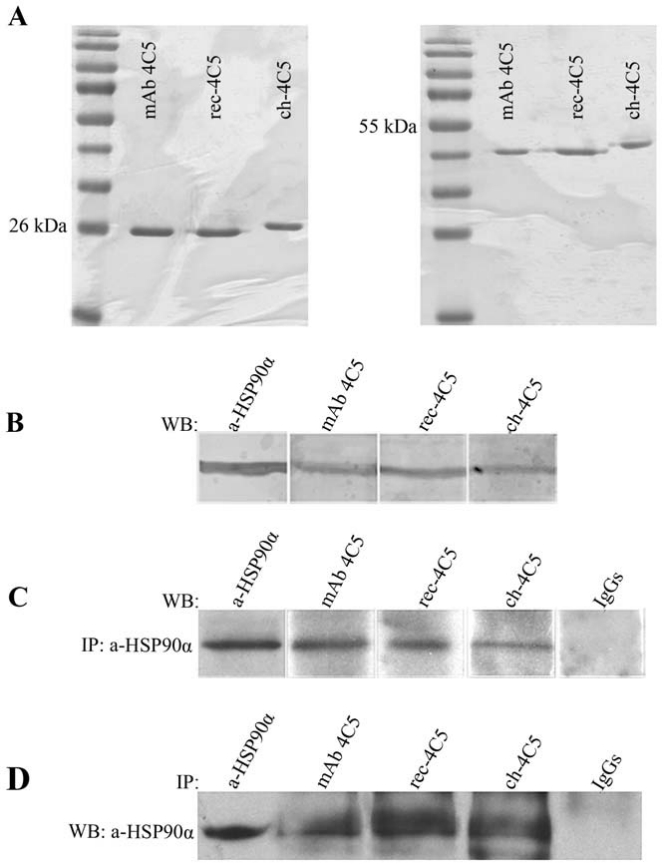
Electrophoretic mobility and specificity of antibodies. **A**. *Left panel:* SDS-PAGE of purified antibodies under reducing conditions followed by Coomasie Brilliant Blue-R staining revealed in all cases an approximately 25 kDa band corresponding to the L-chain. *Right panel:* Under non-reducing conditions the antibodies are shown to migrate as a L-chain dimer. **B**. Western blot of MDA-MB-453 cell lysates using mAb 4C5, rec-4C5, ch-4C5 and a commercial anti-HSP90α antibody, serving as positive control. In all cases a single 90 kDa immunoreactive band corresponding to HSP90 is observed. **C**. Immunoprecipitation in MDA-MB-453 cell lysates with anti-HSP90α, followed by immunoblotting with either the murine or the recombinant antibodies. In all cases a single immunoreactive band is observed. **D**. Reverse immunoprecipitation experiments in MDA-MB-453 cell lysates using mAb 4C5, rec-4C5 and ch-4C5, followed by western blot with anti-HSP90α. In all cases a single immunoreactive band is observed indicating that both recombinant antibodies recognize HSP90.

Both rec-4C5 and ch-4C5 were expressed in the bacterial periplasmic space and purified as described in Materials and Methods. The electrophoretic motilities of the purified antibodies were tested under reducing and non-reducing conditions and were found to be similar to the corresponding motility of the original mAb 4C5 antibody (Fig. 3A).

### ch-4C5 specifically recognizes HSP90

In order to explore the specificity of the antibodies, western blot analysis was performed in MDA-MB-453 breast cancer cell lysates using a commercial polyclonal anti-HSP90α antibody (Chemicon International, USA), mAb 4C5, rec-4C5 and ch-4C5. In all cases a single identical immunoreactive band was observed (Fig. 3B), confirming that both rec-4C5 and ch-4C5 retain the specificity of the paternal mAb 4C5. This result was further confirmed by immunoprecipitation experiments performed in pre-cleared MDA-MB-453 cell lysates using anti-HSP90α, followed by immunoblotting with mAb 4C5, rec-4C5 or ch-4C5. In all cases, a single immunoreactive band was observed, indicating that the chimeric L-chain specifically recognize HSP90 (Fig. 3C). The same result was obtained when immunoprecipitation was performed using mAb 4C5, rec-4C5 and ch-4C5 followed by western blot with the anti-HSP90α antibody (Fig. 3D). In all experiments an irrelevant mouse IgG was used as negative control.

### ch-4C5 is cell-impermeable and does not affect the function of intracellular HSP90

**Figure 4:**
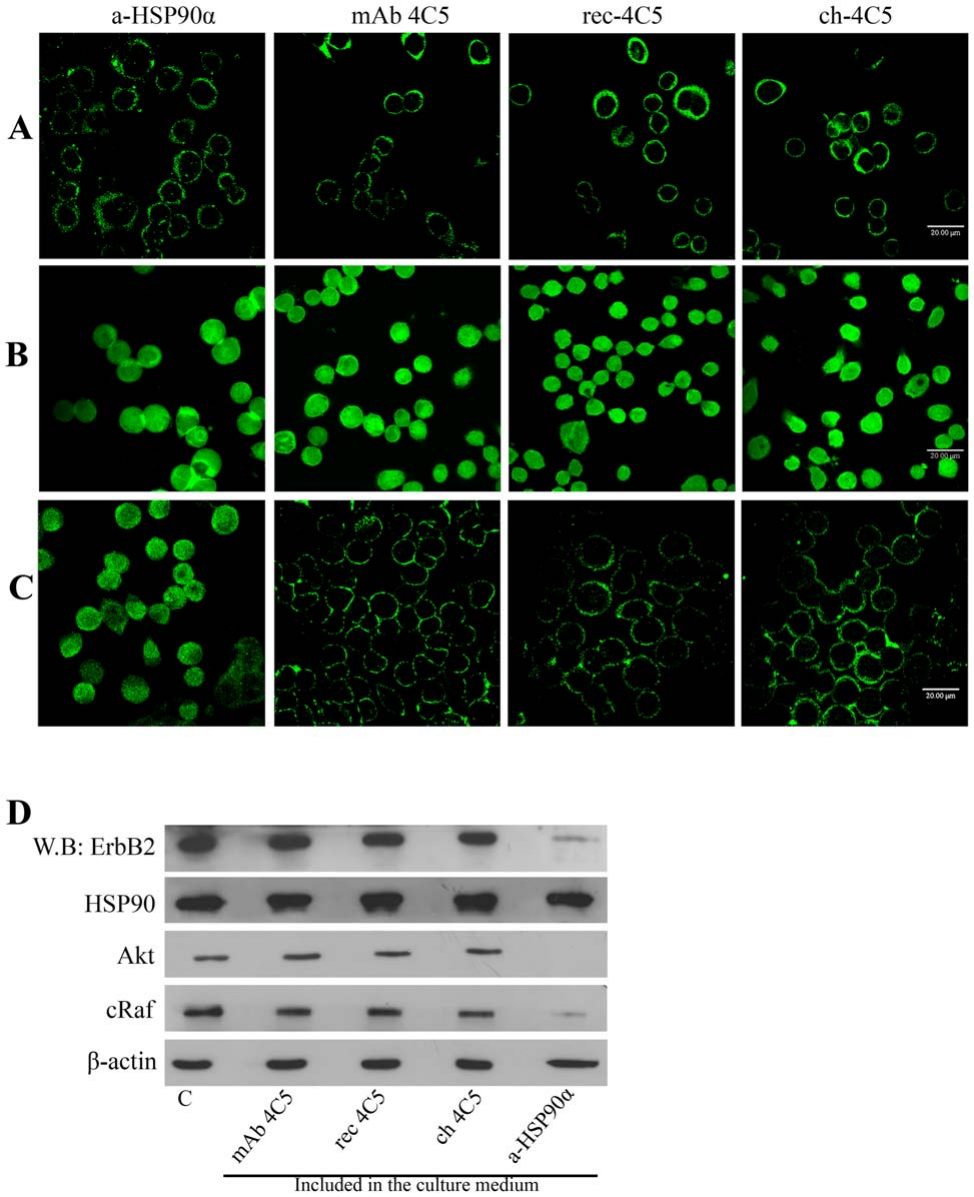
ch-4C5 binds to the surface pool of HSP90 and is not internalized by MDA-MB-453 cells. **A**. Immunofluoresence labelling of MDA-MB-453 cells using both the rec-4C5 and ch-4C5. The punctuate immunolabeling indicates the surface pool of HSP90. Negative controls were performed using an antibody against the intracellular protein β tubulin (data non shown). Scale bar: 20 µm **B**. Immunofluoresence detection of intracellular HSP90 in fixed MDA-MB-453 cells, permeabilized with 0.1% Triton X-100. Similarly to the murine antibody, rec-4C5 and ch-4C5 recognize the intracellular pool of HSP90. Scale bar: 20 µm **C**. For detection of antibody internalization, live cells were incubated with the antibodies for various time intervals, then fixed, permeabilized and fluorescently labelled. No internalization of mAb4C5, rec-4C5 and ch-4C5 is observed even after 24 h of incubation. In contrast the anti-HSP90α antibody could be detected intracellularly after 24 h of incubation. Scale bar: 20 µm **D**. MDA-MB-453 cell lysates, treated with mAb 4C5, rec-4C5, ch-4C5 and anti-HSP90α were analyzed by western blot using antibodies against ErbB2, Akt, cRaf and HSP90α. Actin served as a loading control. Presence of mAb 4C5, rec-4C5 and ch-4C5 did not affect the levels of the kinases compared to controls. In contrast the cell permeable anti-HSP90α antibody significantly reduced the levels of ErbB2, Akt and cRaf.

In order to investigate whether ch-4C5 binds to surface HSP90, unfixed MDA-MB-453 cells were incubated with either rec-4C5 or ch-4C5 while in culture. Thus, the antibodies had access only to the external surface of the cells. Following incubation, cells were processed for indirect immunofluoresence using a fluorescently-labelled secondary antibody. The observed typical punctuate immunostaining confirmed the cell surface labeling (Fig. 4A). Similar results were obtained when using anti-HSP90α and mAb 4C5 (Fig. 4A). It is noteworthy that similarly to mAb 4C5, ch-4C5 as well as rec-4C5 also recognize the intracellular pool of HSP90 as demonstrated by immunofluoresence after fixation and permeabilization of MDA-MB-453 cells (Fig. 4B). Finally, the binding of antibodies to live MDA-MB-453 cells was monitored at various time intervals. As shown in Fig. 4C, similarly to mAb 4C5, rec-4C5 and ch-4C5 were not internalized and remained bound on the cell surface for up to 24 h in culture.

The significance of ch-4C5 cell-impermeability was investigated by monitoring the levels of a subset of intracellular HSP90 client proteins. To date more than 100 intracellular HSP90 client proteins have been identified. Cell-permeable HSP90 inhibitors induce the degradation of these proteins because their stability depends on intracellular HSP90 function [3]. Since our data suggested that mAb 4C5 and subsequently rec-4C5 and ch-4C5 are cell impermeable, we examined their ability to impact the stability of three well-characterized HSP90 client proteins, Akt, cRaf and ErbB-2. Following incubation of MDA-MB-453 cells with different concentrations of mAb 4C5, rec-4C5 and ch-4C5, the levels of Akt, cRaf and ErbB2 were monitored by western blotting. As shown in Figure 4D these antibodies did not affect the steady-state levels of any of the kinases studied. In contrast when the cells were incubated with the cell permeable anti-HSP90α, expression levels of these kinases were significantly reduced (Fig. 4D).

### ch-4C5 inhibits cancer cell invasion

**Figure 5:**
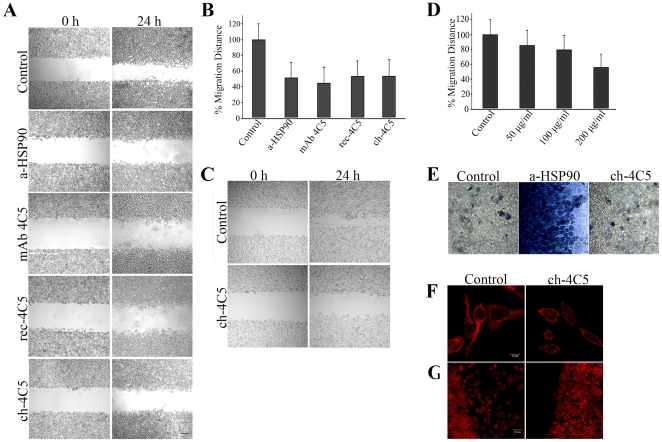
ch-4C5 inhibis cancer cell invasion in *vitro*. **A**. Wound healing assay. Photographs represent phase-contrast images obtained at zero time (left panel) and at 24 h (right panel) after scratch formation, showing MDA-MB-453 cell migration either in control cultures or cultures including anti-HSP90α, mAb 4C5, rec-4C5 or ch-4C5. Scale bar: 200 µm. **B**. Quantitative effect of antibodies on the closure of the wound. Addition of 200 µg/ml of anti-HSP90α and mAb 4C5 in the culture medium resulted in a 48% and 55% reduction of wound closure, respectively when compared to control cultures that were considered as resulting in 100% wound closure. Addition of 200 µg/ml rec-4C5 and ch-4C5 in the culture medium resulted in a 46% and 46% inhibition of wound closure, respectively. Statistical significance of differences was assessed by Student's t test. The presence of anti-HSP90α, mAb 4C5, rec-4C5 or ch-4C5 had a statistically significant effect on the wound closure (p<0.01 in each case). **C**. Phase-contrast images obtained at zero time (left panel) and at 24 h after scratch formation (right panel), showing B16 F10 melanoma cell invasion in a wound healing assay in the presence of 200 µg/ml of ch-4C5. Scale bar: 200 µm. **D**. Quantitative effect of increasing concentrations of ch-4C5 on the invasion level of B16 F10 melanoma cells. Presence of 50 µg/ml ch-4C5 resulted in 15% inhibition of invasion, while addition of 100 µg/ml and 200 µg/ml ch 4C5 resulted in 21% and 43% inhibition of migration, respectively when compared to control cultures that were considered as resulting in 100% wound closure. **E**. Visualization of dead cells using trypan blue dye. In ch-4C5 treated cultures, the cell death incidence is similar to that observed in the control cultures. In contrast, in the cultures treated with the anti-HSP90α antibody, a much greater number of cells are stained with Trypan blue, indicating an increased incidence of cell death Scale bar, 30 µm. **F**. Control and ch-4C5 treated cells were fixed permeabilized and stained with fluorescently labelled phalloidin. Scale bar 40 µm **G**. Higher magnification showing phalloidin staining (F-actin). Ch-4C5 effectively blocks spreading of lamellipodia. Scale bar: 16 µm.

Previous studies have shown that mAb 4C5 inhibits MDA-MB-453 breast cancer and B16 F10 melanoma cell invasion in a wound healing assay [11], [21]. In order to confirm that ch-4C5 exhibits the same functional property, we performed *in vitro* wound healing assays using MDA-MB-453 and B16 F10 cancer cells. Control cultures were grown either in culture medium alone, or in culture medium containing 200 µg/ml of the irrelevant BM88 antibody. No statistically significant difference was observed between the two types of controls used. The mean value of the two types of control was considered as 100% of wound closure. As shown in Fig. 5A,B the presence of ch-4C5 in the culture medium significantly reduced the extend of MDA-MB-453 cancer cell invasion within the migration gap after 24 h, as compared to control cultures. More specifically, the presence of ch-4C5 resulted in a 46% inhibition of wound closure, which was similar to that obtained when the anti-HSP90α, as well as mAb 4C5 and rec-4C5 were included in the culture medium (Fig. 5A,B). The bars in Fig. 5B represent the average of three independent experiments ±SEM. Within a single experiment, each condition was tested in triplicate. Similar results were obtained using B16 F10 melanoma cells and increasing concentrations of ch-4C5 which yielded a dose dependent inhibition of cell invasion (Fig. 5C,D). It is important to note that in the wound healing assay the effect of ch-4C5 is directed towards cell invasion and not cell proliferation as judged by an independent MTT assay (data not shown). This is in accordance with previously published data demonstrating that the presence of mAb 4C5 in the culture medium of B16 F10 [11] and MDA-MB-453 [21], does not affect cell proliferation, since low BrdU incorporation was observed with no apparent differences in the absence or presence of the antibody.

At this point it should be noted that the inhibitory effect of the anti-HSP90α antibody on the MDA-MB-453 invasion rate was in great part due to increased cell death as judged by trypan blue staining, which selectively colours dead cells (Fig. 5E). In contrast, when cultures were treated with mAb 4C5 and ch-4C5, the incidence of cell death was similar to that observed in the control cultures (Fig. 5E). This result further supports that ch-4C5, in contrast to the cell-permeable anti-HSP90α antibody, is cell-impermeable and does not affect the intracellular pool of HSP90, which is important for cell survival.

Finally these cultures were examined with respect to actin re-arrangement dynamics using fluorescently labelled phalloidin. MDA-MB-453 cells exposed to ch-4C5 were less spread compared to cells in control cultures, and their morphology was indicative of non-motile cells. Furthermore, when visualized at a higher magnification, the lamellipodia in treated cultures were less developed and less spread out compared to lamellipodia in control cultures (Fig. 5 F,G). These results are in accordance with previously published data regarding the effect of mAb 4C5 on actin re-arrangement and lamellipodia development [21].

### ch-4C5 reduces the MDA-MB-453 metastatic deposition into the lungs of SCID mice

**Figure 6:**
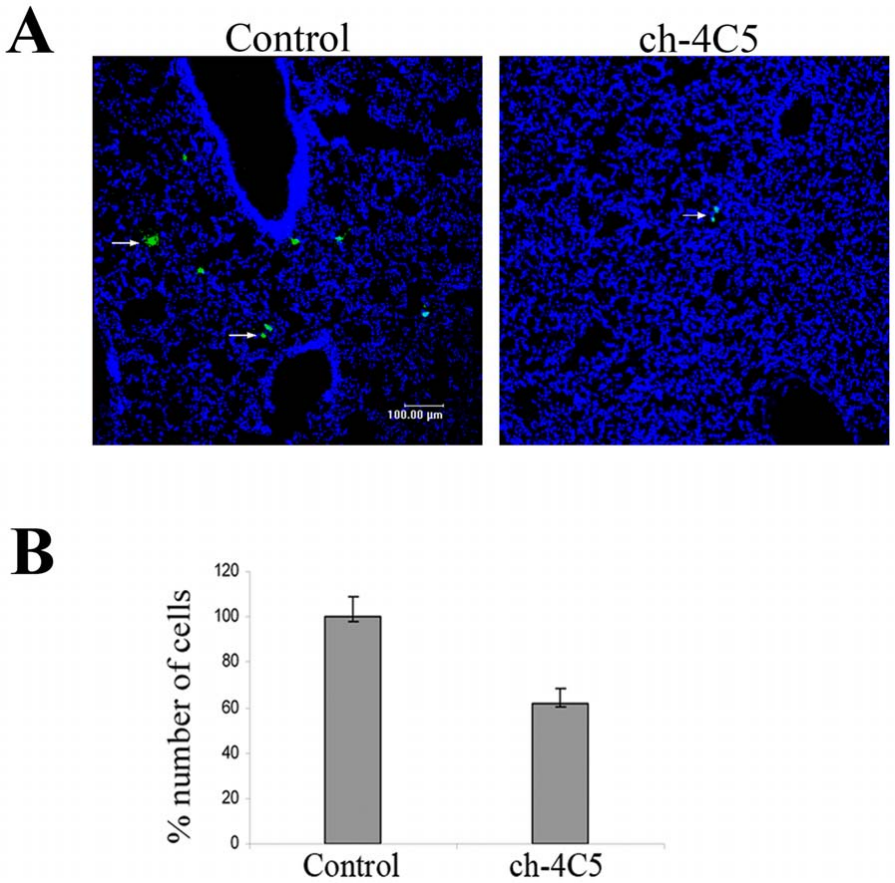
ch-4C5 inhibits the metastatic deposition of MDA-MB-453 cancer cells into the lungs of SCID mice. Control or ch-4C5 treated MDA-MB-453 cells were labeled with the fluorescent dye DiO and injected into SCID mice as described in Materials and Methods. Evaluation of metastatic deposits was performed several hours later. A. Representative cryosections of the lungs of control and ch-4C5 treated mice. The arrows show MDA-MB-453 cells stained with DiO present in the lung tissue. A significant decrease in the deposition of cancer cells was observed in the lungs of ch-4C5 treated mice. Scale bar: 100 µm **B**. Quantitative effect of ch-4C5 on the metastatic deposition of MDA-MB-453 cells into the lungs, showed a 38% inhibition when compared to control animals. The bars represent the average of two independent experiments ±SEM. Statistical significance of differences was tested by Student's t test (p<0.01).

We next sought to investigate the *in vivo* effect of ch-4C5 on the metastatic behaviour of MDA-MB-453 cancer cells. For this purpose, MDA-MB-453 cells were pre-treated or not with 200 µg/ml ch-4C5 and then labelled with the fluorescent dye DiO and injected intravenously into SCID mice via the tail vein. Twelve hours after the injection, mice were euthanized and the metastatic deposits of MDA-MB-453 cells were traced and evaluated in the lungs of both the control and the ch-4C5-treated groups. As shown in Fig. 6A, a significant decrease in the deposition of MDA-MB-453 cells was detected in the ch-4C5 treated mice as compared to the control animals. More specifically, quantification of the metastatic deposit formation showed a 38% inhibition in the ch-4C5 treated mice when compared to control mice (Fig. 6B).

## Discussion

In this study, we describe the cloning and sequencing of an anti-HSP90 murine monoclonal antibody, named mAb 4C5. More importantly, we demonstrate that mAb 4C5 is not a conventional IgG molecule, but instead it is completely devoid of a H-chain and only consists of a kappa light chain dimer. This finding was initially supported by SDS-PAGE electrophoresis under denaturing and non-denaturing conditions, demonstrating that mAb 4C5 migrates unconventionally at approximately 26- and 50-kDa, respectively. Additionally, when total RNA isolated from the mAb 4C5-producing hybridoma cell line was subjected to northern blot hybridization using an IgG1 H-chain cDNA radio-labeled probe, no radioactivity could be detected. In agreement with the above results, when we attempted to amplify the H-chain cDNA using universal mouse H-chain primers we could not isolate a H-chain product in all conditions tested. Finally, the unusual nature of mAb 4C5 was confirmed beyond any doubt, since the recombinant kappa L-chain expressed in bacteria was shown to retain all the properties of the paternal antibody, including antigen binding and *in vitro* inhibition of cancer cell invasion.

This monoclonal antibody was originally produced by immunization of mice with a brain-derived membrane fraction of 15 day-old rat embryos [17], and it was shown to specifically recognize and inhibit the function of surface HSP90 during cell migration processes [8]. Additionally, mAb 4C5 was shown to significantly reduce the rate of invasion and metastasis of cancer cells. More specifically, it was demonstrated that this antibody inhibits melanoma cell invasion and metastasis [11] as well as the interaction of surface HSP90 with the extracellular domain of ErbB-2, leading to impaired downstream signalling and subsequently reduced rate of *in vitro* breast cancer cell invasion [21]. Even more recently, Stellas et al. [14] provided *in vitro* and *in vivo* evidence showing that mAb 4C5 indirectly inhibits the activation of pro-gelatinases MMP-2 and MMP-9, necessary for cancer cell invasion, extravasation and metastasis. These combined data further supported the idea that mAb 4C5 could be useful as a cancer therapeutic and oriented our studies towards the determination of the aminoacid sequence and recombinant expression of this antibody.

It is well known that murine antibodies have limited use for *in vivo* therapy in humans because of their immunogenicity. In multiple instances this problem has been overcome using genetic engineering approaches to produce chimeric mouse-human and fully humanized antibodies. Taking into consideration the unconventional nature of mAb 4C5 in combination with the fact that during the humanization process the antibody affinity is frequently reduced, we next reconstituted the murine mAb 4C5 into a functional mouse-human chimeric version that, like the paternal antibody, binds to surface HSP90, is cell impermeable and inhibits cancer cell invasion. The chimeric antibody which was engineered by replacing the Cκ- region of the paternal murine antibody with the corresponding human Cκ-region was shown to retain the specificity and affinity of the paternal mouse antibody.

It has been a generally accepted concept that the antibody molecule requires both the H- and L-chains for its full activity [27]. Moreover, the H-chain is believed to be the predominant contributor to the free energy of antigen binding while the contribution of the L-chain is supposed to be limited [28], [29]. The latter idea is further supported by the fact that cameloids possesses a class of fully functional antibodies completely lacking L-chains and consisting of H-chain dimers [30], [31]. In context to these findings, H-chains alone were shown to interact with a variety of antigens in a specific manner (albeit with lower affinity than the intact antibodies), which has led to the application of single domain antibodies derived from the H-chains [32] in research, biotechnology as well as human therapeutics. In the past, there have been sporadic reports of antigen binding by L-chains. The first examples of free inmmunoglobulin light chains concerned the so called Bence-Jones proteins. These were reported as L-chain dimers expressed by multiple myeloma cells, collected from the urine of human patients [33]. Furthermore, large amounts of L-chains were found to accumulate in the extracellular fluids and tissues of patients with L-chain secreting tumors [34]. A kappa light chain dimer with CD4 antigen specificity derived from a hybridoma cell line was described in 1987 by Ledbetter et al. [35] and in 1994 Mei Sun et al [36] reported that a purified L-chain from a monoclonal antibody against vasoactive intestinal polypeptide (VIP) displayed sequence-specific and high affinity binding to VIP. Accordingly, Nishimura et al. [37] reported the production of a recombinant λ light chain exhibiting a significantly higher activity of binding to the antigen compared with the intact antibody. Furthermore, these authors presented evidence that this recombinant light chain could serve as a potentially useful vehicle for clinical use such as radio-immunoimaging and radio-immunotherapy of lung cancers. It has been also reported that antibody L-chain dimers produced by a mouse hybridoma react with human melanoma tissues [38]. In 1998 Pereira et al. [39] showed that a single L-chain variable sequence contains all the determinants necessary for cardiolipin binding, with an affinity similar to that of the intact antibody. More recently, Dubnovitsky et al. [40] reported that the recombinant V_L_-domain of a monoclonal antibody against ferritin preserved its antigen binding function with an affinity comparable to that of the full-length parental antibody.

In the present work we have demonstrated that ch-4C5 is completely devoid of a heavy chain and consists of a L-chain dimer. ch-4C5 has a high specific activity as demonstrated by immunoblotting and immunofluoresence experiments using breast cancer cells. Furthermore and similarly to the paternal antibody ch-4C5 exhibits function-blocking properties as judged by the *in vitro* wound healing assay. Finally, using an *in vivo* assay we have demonstrated that ch-4C5 reduces the metastatic deposition of MDA-MB-453 breast cancer cells into the lungs of SCID mice.

These results provide a new perspective for the clinical application of L-chain antibodies in cancer therapeutics. Recombinant L-chain antibodies have a number of advantages over intact antibodies and V_H_ antibodies when intended for human therapeutics, i.e relatively facile and reproducible production, quality control procedures, and faster clearance from the circulation. Finally from a clinical point of view, surface HSP90 provides a novel and very promising extracellular drug target for the effective treatment of metastatic cancer. In this context, the ability of ch-4C5 to selectively inhibit the function of the surface pool of HSP90, as indicated by the *in vitro* and *in vivo* data presented in the current work, without interfering with HSP90 intracellular function, renders this antibody fragment a potential cancer therapeutic.

## Materials and Methods

### Ethics Statement

All animal studies were conducted according to the national and international guidelines and were specifically approved by the Ethical Committee of the Hellenic Pasteur Institute (Permit No: K/533, Veterinary Directorate District of Attiki). SCID mice were originally purchased from Jackson Laboratory, bred and maintained under specific pathogen free conditions at the Experimental Animal Unit of the Hellenic Pasteur Institute. All of the experiments with animals were performed in accordance with the guidelines approved by the Ethical Committee of the Hellenic Pasteur Institute.

### RNA and DNA manipulations

Total RNA was isolated from 10^7^ hybridoma cells [17], using the ‘Absolutely RNA Mini Prep kit’ (Stratagene). For Northern blot analysis, 25 ug RNA was separated by agarose electrophoresis, transferred to Zeta-Probe (Biorad) and hybridized with a 1.2-kb H-chain IgG1α cDNA probe as previously described [41]. For amplification of the V_H_ and V_L_ antibody fragments, specific first strand cDNAs were synthesized using the ‘1^st^ Strand cDNA Synthesis kit for RT-PCR’ (Roche), according to the manufacturer's instructions. The cDNAs were subsequently amplified by PCR, using specific mouse immunoglobulin primers [25]. The amplified L-chain cDNA fragment was inserted into the SacI/XbaI restriction sites of the pComb3H phagemid (generous gift of Drs. C. F. Barbas and D. R. Burton, The Scripps Research Institute, La Jolla, CA) [25] and sequenced in both directions at the sequence facility of the Institute of Molecular Biology and Biotechnology (IMBB). The germline counterpart of the rearranged V_L_ sequence was analyzed using the National Center for Biotechnology Information IgBLAST server (http://www.ncbi.nlm.nih.gov/igblast/) and sequence was aligned using ClustalW software.

### Construction of mouse-human ch-4C5

The mouse-human chimeric antibody was constructed by fusing the mAb 4C5 V_L_ cDNA to a human Cκ gene segment. Briefly, the kappa light chain of mAb 4C5 was subcloned into pBluescript SK-plasmid, and then the BsgI/XbaI fragment containing the mouse Cκ region was replaced by the BsgI/XbaI restriction fragment containing the human Cκ. Finally, the chimeric kappa L-chain gene was inserted into the SacI/XbaI sites of pComb3H. All DNA manipulations were performed according to Sambrook [42].

### Expression, purification and electrophoretic analysis of recombinant L-chains

Soluble recombinant antibody L-chains were produced from individual bacterial colonies as described previously [25] and the periplasmic fractions were extracted according to Charlton [43] and purified by affinity chromatography using a KappaSelect column (GE Healthcare) on an FPLC AKTA system (Amersham Biosciences, Piscataway, NJ), according to the manufacturer's instructions. The electrophoretic motilities of antibodies were analyzed under reducing and non-reducing conditions and visualized either with Coomassie Brilliant Blue-R stain or by immunoblot using specific secondary HRP-conjugated antibodies.

### Preparation of cell lysates, immunoprecipitation and western blot analysis

MDA-MB-453 cell lysates were obtained, quantified and processed for immunoprecipitation as previously described [21]. Bound proteins were analyzed by gel electrophoresis followed by western blot and antibody complexes were detected using DAB (Sigma) or/and an ECL chemiluminescence reagent (Amersham), as described by the manufacturers. For all immunoprecipitation experiments, negative controls were performed using irrelevant IgGs.

### Cell cultures and immunofluorescence

MDA-MB-453 breast cancer (ATCC Cell Biology Collection) and B16 F10 melanoma (ATCC Cell Biology Collection) cell lines were maintained in RPMI and DMEM, respectively supplemented with 10% fetal bovine serum (FBS). For immunofluorescence studies, MDA-MB-453 cells were plated on poly-L-lysine coated coverslips at a density of 5×10^4^ cells/well in 48-well plates, fixed and processed as previously described [17]. Live MDA-MB-453 cells were labelled by indirect immunofluorescence as previously reported [8]. Alexa546-labeled phalloidin (Molecular Probes, Eugene, OR) was used to visualize F-actin. For all experiments, controls were performed by omitting the primary antibodies or by using an IgG2a mAb against the unrelated neuronal protein BM88 [44]. Immunofluorescence was analyzed by confocal microscopy using a Leica TSC confocal microscope.

For the antibody internalization assay, MDA-MB-453 cells were incubated with the antibodies while in culture for various time intervals. The cells were then washed and fixed. For detection of possible internalization of the antibody, cells were permeabilized with 0.1% Triton X-100 and subsequently incubated with Alexa488-conjugated secondary antibody (Molecular Probes, Eugene, OR). For all experiments, controls were performed as described above.

### Wound healing assay

The assay was performed as previously described [21]. Briefly, MDA-MB-453 and B16 F10 cells were plated in 48-well plates at a density of 1×10^5^ and 2.5×10^5^ cells/well, respectively. After 24 h the medium was changed to serum free and 16 h later a cell free area was generated by scratching the cell monolayer with a sterile yellow pipette tip, thus resulting in the formation of an approximately 1 mm-wide cell-free area. Immediately after scratching, the medium was replaced with fresh, containing anti-HSP90α, mAb 4C5, rec-4C5 or ch-4C5. All agents were maintained in the culture for the duration of the assay. Control cultures were grown either in culture medium alone or in culture medium containing the BM88 antibody. Migration of cancer cells within the gap was monitored microscopically at given time intervals, using a Leica DM IL inverted microscope, equipped with a LEICA DM300 video camera. Migration distance was estimated by acquiring and analyzing digital images, using Image Pro Plus analysis software. Wound closure in the control cultures was considered as 100% and wound closure in the antibody treated cultures was estimated proportionally compared to the control cultures. Statistical analysis was performed using Student's t-test.

### Trypan blue staining

At the end point of the wound-healing assay, MDA-MB-453 cells were incubated for 5 min with 0.4% trypan blue in PBS, washed and visualized using a Leica microscope.

### MDA-MB-453 cell metastatic deposit formation in the lung

The *in vivo* metastatic deposit formation assay was performed as previously described [14], [45]. Briefly, MDA-MB-453 cells were pre-incubated with either 200 µg/ml ch-4C5 or the unrelated BM88 antibody for 2 hours, washed and incubated with DiO for an additional 1 hour. Cells were then washed again, trypsinized and counted. Twenty 8–10-week-old female SCID mice were divided into two equal groups: the control group injected with non-treated cells or BM88- treated cells and the ch-4C5 treated group The animals were euthanized 12 hours later and their lungs were completely washed from the remaining blood, by pumping 200 ml of saline buffer with the use of a peristaltic pump connected to the left ventricle of the heart. This procedure ensures that all cancer cells which are not attached, either on the inner surface of the blood vessels or on the lung tissue, are removed. Finally the lungs were perfused with 4% formalin solution and then embedded in OCT solution and processed for sectioning. Each cryosection was counter stained with TOPRO-3 and visualised using a confocal microscope. The same experiment was performed twice with similar results. Statistical analysis was performed using the Student's t-test.
